# SOX2 regulates multiple malignant processes of breast cancer development through the SOX2/miR-181a-5p, miR-30e-5p/TUSC3 axis

**DOI:** 10.1186/s12943-017-0632-9

**Published:** 2017-03-14

**Authors:** Kuancan Liu, Fuan Xie, Anding Gao, Rui Zhang, Long Zhang, Zhangwu Xiao, Qiong Hu, Weifeng Huang, Qiaojia Huang, Baoshun Lin, Jian Zhu, Haikun Wang, Jianwen Que, Xiaopeng Lan

**Affiliations:** 1 Institute for Laboratory Medicine, Fuzhou General Hospital, PLA, Fuzhou, 350025 Fujian People’s Republic of China; 2 Department of Medicine, Columbia University Medical Center, New York, 10032 NY USA; 3 Dong fang Hospital, Xiamen University, Fuzhou, 350025 Fujian People’s Republic of China; 4 Life Science Institute, Zhejiang University, Hangzhou, 310058 Zhejiang People’s Republic of China; 5 Emergency Department of the 476 Hospital, Fuzhou General Hospital, PLA, Fuzhou, 350002 Fujian People’s Republic of China; 6 Fuzhou General Hospital Clinical Medical School, Fujian Medical University, Fuzhou, 350025 People’s Republic of China; 7 Medical College, China Three Gorges University, Yichang, 443002 Hubei People’s Republic of China; 8 Department of Microbiology and Immunology, University of Rochester, Rochester, 14642 NY USA; 9 Institute Pasteur of Shanghai, Chinese Academy of Sciences, Shanghai, 200031 People’s Republic of China

**Keywords:** Sox2, Breast cancer, Tumourigenesis, miRNA

## Abstract

**Background:**

High levels of SOX2 protein are correlated with increased dissemination of breast cancer. However, the underlying molecular mechanisms are not fully understood.

**Methods:**

In this study we investigate the role of SOX2 in breast cancer metastasis using multiple in vitro and in vivo assays including cell culture, shRNA-mediated knockdown, wound healing, colony formation, transwell chamber, xenograft and tail vein injection. Moreover, western blot, immunostaining, microarray and real-time PCR were used to determine the change of protein and miRNA levels. Luciferase assays were also used to evaluate activity which TUSC3 is a target of miR-181a-5p and miR-30e-5p, and the clinical survival relevance was analyzed by Kaplan-Meier analysis.

**Results:**

We identified a novel pathway involving SOX2 regulation of microRNAs to control the proliferation and migration of breast cancer cells. shRNA-mediated knockdown of SOX2 inhibits breast cancer cell expansion and migration. More importantly, we found that these changes are accompanied by significant reduction in the levels of two microRNAs, miR-181a-5p and miR-30e-5p. Overexpression of these two microRNAs leads to reduced protein levels of Tumor Suppressor Candidate 3 (TUSC3) in breast cancer cells; mutations of the potential binding sites in the 3’-UTR of TUSC3 abrogate the inhibitory effects of the microRNAs. We further found that upregulation of TUSC3 expression leads to reduced proliferation and migration of breast cancer cells. In human breast cancer samples the levels of TUSC3 protein are inversely correlated with those of SOX2 protein.

**Conclusions:**

Taken together, our work reveals a novel SOX2-mediated regulatory axis that plays critical roles in the proliferation, migration and invasiveness of breast cancer cells. Targeting this axis may provide beneficial effect in the treatment of breast cancer.

**Electronic supplementary material:**

The online version of this article (doi:10.1186/s12943-017-0632-9) contains supplementary material, which is available to authorized users.

## Background

Breast cancer is a leading cause of cancer-related death in women worldwide. Despite the significant progress that has been made in cancer treatment and early detection, approximately 30 to 70% of breast cancer patients die from breast cancer recurrence or metastasis [1]. Therefore it is important to investigate the mechanisms underlying cancer cell proliferation and metastasis-related cellular activities such as cell migration and invasion. Genetic mutations or epigenetic modifications that provide growth advantage are critical for tumour initiation and progression. In breast cancer, activation mutations of genes such as *EGFR*, *HER2* have been found to transmit signals into the nucleus to up-regulate the transcription of downstream genes, promoting cancer cell growth [2–4]. However, little is known about the mediators in the nucleus that are involved in gene regulation. Interestingly, the transcription factor SOX2 has recently been associated with breast cancer formation and metastasis [5]. SOX2 belongs to the sex determining region Y (SRY)-like box (SOX) gene family, members of which are pivotal for normal development and maintenance of stemness [6–11], cell proliferation and differentiation [12]. Emerging studies demonstrated that the SOX2 protein is also involved in tumourigenesis [13–15]. High levels of SOX2 closely correlate with multiple processes during tumour development, including initiation [16], maintenance [17, 18], invasion and metastasis [19–21]. Accordingly, aberrant *SOX2* expression was detected in multiple types of cancers at different stages. We and others have shown high levels of SOX2 in lung cancer, esophageal squamous cell carcinomas (ESCC) and ovarian cancer [13, 22–26]. Interestingly, high levels of SOX2 proteins have also been found in 19% of breast cancer patients [5]. Moreover, SOX2 expression has been linked to tamoxifen resistance and relapse in breast cancer treatment [27]. Nevertheless, the mechanisms by which high levels of SOX2 regulate the progression and metastasis of breast cancer remain largely unexplored.

MicroRNAs (miRNAs) are small, noncoding RNAs that are important for the regulation of gene expressions at the post-transcriptional level. miRNAs are involved in almost all key cellular activities such as proliferation, differentiation and migration [28]. Dysregulation of miRNAs is also involved in the initiation and progression of human cancers including breast cancer [29–31]. For example, miR-124 is down-regulated in breast cancer cell lines. Ectopic expression of miR-124 reduces the protein levels of Myc and phospho-Rb while up-regulating p27 in the breast cancer cell line MDA-MB-231 [32], suggesting that miRNAs function as a tumour suppressor. In this study we examined the molecular mechanisms used by SOX2 in the proliferation and migration of breast cancer cell lines and identified that SOX2 regulates two miRNAs (miR-181a-5p and miR30e-5p) which in turn influence the expression of a common downstream target TUSC3. Importantly, inversed expression of SOX2 and TUSC3 is associated with poor prognosis of a subpopulation of breast cancer patients. Therefore we demonstrate here a clinically relevant axis which involves SOX2/miRNAs/TUSC3 in breast cancer development.

## Methods

### Cell lines and mice

ZR7530 (Cat NO: CRL-1504™) and MDA-MB-231 (Cat NO: HTB-26™) breast cancer cell lines were purchased from ATCC (Manassas, VA, USA), Cell line authentication was conducted by short tandem repeat analysis, and mycoplasma contamination in cell lines was tested using multiplex PCR. They were infected with lentivirus carrying *pLK0.1SOX2* (SOX2 knockdown plasmid), *pCDH-SOX2-IRES-GFP* (SOX2 overexpression plasmid), *pLK0.1*Scramble (control plasmid) [33], and *pCDH-TUSC3-IRES-GFP* (TUSC3 overexpression plasmid, primers used for construction were shown in Additional file 1: Table S1). Puromycin (Santa Cruz Biotechnology, Cat No: sc-108071, Dallas, TX, USA) selection was used to establish cell lines stably expressing these constructs. Cell lines were cultured in Dulbecco’s Modified Eagle Medium (DMEM, HyClone, Cat No: SH30022.01B, Beijing, China) with 10% foetal bovine serum (FBS, Gibco, Cat No:10270-106, Grand Island, NY, USA). Nude mice at 6-weeks old were purchased from SLRC Laboratory Company (Shanghai, China).

### Western blot analysis

Western blot was performed as previously described [34]. PVDF membrane was used for protein transfer and probed with antibodies against human SOX2 (polyclonal rabbit anti-SOX2, SEVEN HILLS, Cat No: WRAB-1236, Cincinnati, USA), human β-actin (mouse anti-β-actin, Beyotime, Cat No: AA128, Nantong, China), CDK4 (BBI, Cat No: AB20396b, Shanghai, China), CDK6 (BBI, Cat No: AB20398a, Shanghai, China), CCND1 (BBI, Cat No: AB60236b, Shanghai, China), TUSC3 (Novus Biologicals, Cat No: NBP1-55630, Littleton, USA). HRP-conjugated goat anti-rabbit IgG (Abcam, Cat No: ab136817, Cambridge, MA, USA) and HRP-conjugated goat anti-mouse IgG (ZSGB-Bio, Cat No: ZB-2305, Beijing, China) were used as secondary antibodies to detect the proteins. The density of protein band was quantified with Image-Pro Plus 6.0 software, and the ratio of target protein to housekeeping protein, which reflects the change of expression level, was calculated.

### Cell proliferation and colony formation assays

ZR7530 or MDA-MB-231 cells with genetic manipulation (knockdown or overexpression) were seeded in 96-well plates. The OD value of cells was measured under 450 nm and 630 nm at 24, 48, 72 and 96 h using the Cell Counting Kit 8 (CCK-8) (Beyotime) assay, and the absorbance difference between 450 nm and 630 nm represents cell proliferation rates. For soft agar colony formation assays, ZR7530 or MDA-MB-231 cells were seeded in 6-well plates and maintained for 2 weeks and colonies were fixed with 4% paraformaldehyde (DingGuo, Cat No: AR-0211, Beijing, China) and stained with 0.5% crystal violet. The number of total colonies and colonies with a diameter greater than 0.5 mm were calculated with Image J (Fiji-win32 software, University of Wisconsin-Madison), and the data were analysed as previously described [35, 36].

### Wound healing assay

1 × 10^6^ ZR7530 or MDA-MB-231 cells (SOX2 knockdown, scramble and TUSC3 overexpression) were seeded in 6-well plates and grown to 90% confluence. Cell monolayers were scraped with a sterile pipette tip. Floating cells were removed and the cultures were maintained in DMEM supplemented with 5% FBS. The wound area was recorded 0 h and 48 h after the scrape. The healing index was calculated and analysed using the formula: (S_0_ − S_n_)/S_0_ × 100%, which represents the ability of cellular migration. S_0_ represents the blank area 0 h after scraping, and S_n_ represents the blank area n h after scraping [37].

### Matrigel invasion assay

Invasion assays were performed in chambers with a 6.5-mm insert in 24-well plates (Corning company, Cat No: 3422, Corning, NY, USA). An 8-μm polycarbonate membrane was coated with 12 μl of ice-cold Matrigel™ Basement Membrane Matrix (BD company, Cat No: 356234, Bedford, MA, USA). Cells (5x10^4^ per well) were added to the upper chamber in 200 μl of the medium containing 5% FBS. The lower chamber was filled with 500 μl of medium containing 20% FBS. The culture was maintained for 24 h. Cells were then fixed in methanol and stained with 1% crystal violet. Cells at the lower side of the filter were counted under a light microscope and dissolved with 50% acetic acid.

### Xenograft studies

1x10^5^ ZR7530 cells were re-suspended in 200 μl of DMEM and subcutaneously injected into the flanks of 6-week old BALB/c nude mice, which were maintained in the SPF environment. Five female nude mice per cohort (SOX2 knockdown and control) were randomly divided to receive cancer cells. Tumour size was measured every 3 days using a Vernier calliper after injection, and tumour volumes were calculated with the formula 0.52 × width^2^ × length [38, 39]. Tumours were collected 7 weeks after cell inoculation.

For lung metastatic experiments, 2 × 10^6^ tumour cells were re-suspended in 100 μl of PBS and injected into BALB/c nude mice via the tail vein. Five female nude mice per cohort, which were also maintained in the SPF environment, received the tumour cells through tail vein. These nude mice were sacrificed 8 weeks after tail vein injection. Lungs were fixed with 4% PFA and sectioned for haematoxylin and eosin (HE) staining to determine the metastatic capabilities of cancer cells. The metastatic capabilities were assessed as previously described [40, 41].

### miRNA microarray assay and real-time PCR

Total RNAs of ZR7530 cells (SOX2 knockdown and control) were extracted and purified using Trizol (Invitrogen, Carlsbad, CA, USA) and miRNeasy kit (Qiagen, Hilden, Germany) according to the manufacturer’s protocol. The human miRNA microarray miRCURY™ LNA Expression Array (v.18.0, Exiqon) was used to detect the level changes after SOX2 knockdown. The low intensity miRNAs were filtered, and original signal intensities were normalised using the Median Normalization Method. The miRNA data were analysed with Significance Analysis of Microarrays (SAM) version 2.1 to generate a list of miRNAs that up-regulate or down-regulate with a fold change ≥1.5 and a *p*-value ≤0.05. Quantitative real-time PCR analysis was used to validate the level changes of miRNA. Additional file 1: Table S2 and Table S3 describe the stem-loop RT primers and specific PCR primers used for quantitative PCR.

### GO and pathway analyses

Differentially expressed miRNAs were clustered using the Cluster software, and we performed miRNA target gene predictions using the microRNA target prediction tools miRBase, TargetScan, and miRanda, and all common target genes which are regulated by two or multiple miRNAs were analyzed with cytoscape program. We also performed Gene Ontology (GO) analysis (http://www.geneontology.org) of the target genes that were predicted using the three databases, and the KEGG database (http://www.genome.jp/kegg/) was selected for pathway analysis based on the target genes of these differentially expressed miRNAs. The significance of the GO terms and Pathway was selected based on a *p*-value cut-off of 0.05.

### Luciferase reporter assays

Luciferase reporter activities were determined using the Dual-Luciferase Reporter Assay System (Promega, Cat No. E1910, Madison, USA). The primers used for obtaining mutated 3’UTR of TUSC3 were described in Additional file 1: Table S4. pGL3 with wild-type or mutated 3’UTR of TUSC3, miRNA mimics and pRL-TK were co-transfected into cells for all luciferase assays, and Renilla luciferase activities were measured to assess the transfection efficiency, sequences of miRNA mimics, inhibitors and relevant controls were shown in Additional file 1: Table S5.

### Statistical analysis

The data represent at least three independent experiments using cells or extracts from a minimum of three separate isolations. Differences between groups were compared using analysis of variance for repeated measures. All statistical analyses were performed using GraphPad PRISM. v5.0 software (San Diego, CA, USA). Data were presented as mean ± SD (standard deviation). Statistical significance between two groups was calculated by unpaired student’s *t*-test, and *P* value < 0.05 is considered significant.

## Results

### SOX2 knockdown leads to reduced proliferation and migration of breast cancer cells

**Fig. 1:**
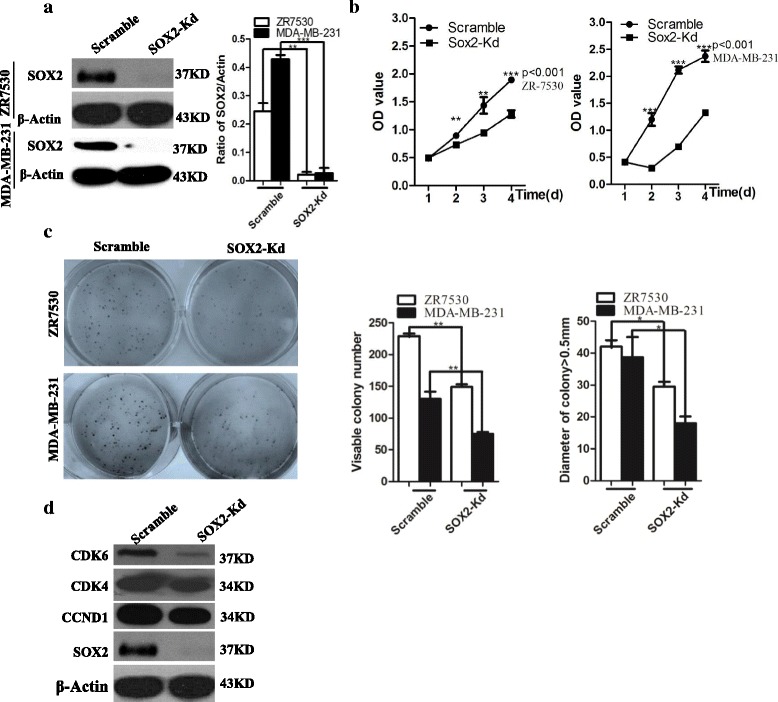
SOX2 knockdown leads to reduced proliferation of breast cancer cells. **a** SOX2 knockdown leads to significant reduction in the levels of SOX2 protein in ZR7530 and MDA-MB-231 cells. **b** SOX2 knockdown inhibits the proliferation of breast cancer cell *lines* ZR7530 and MDA-MB-231. **c** *Decrease* in the levels of SOX2 in breast cancer cells results in reduced colony formation efficiency and colony size. **d** SOX2 knockdown reduces the levels of cell cycle regulators CDK6, CDK4 and Cyclin D1 (CCND1). * indicates *p* < 0.05, ** indicates *p* < 0.01, *** indicates *p* < 0.001 vs control, and the *error bars* indicate the mean ± standard deviation (SD)

The breast cancer cells ZR-7530 and MDA-MB-231 express high levels of SOX2 protein. To test whether SOX2 is required for the proliferation of these cell lines, we performed lentivirus-delivered SOX2 shRNA knockdown in ZR-7530 as previously described [33]. Knockdown leads to up to 85–92% reduction in the levels of SOX2 protein (*p* < 0.01 for ZR-7530 and *p* < 0.001 for MDA-MB-231) (Fig. 1a). More importantly, decreased protein levels are associated with decreased proliferation of cancer cells, especially 96 h after knockdown (*p* < 0.001 for ZR-7530 and *p* < 0.001 for MDA-MB-231) (Fig. 1b). The decrease in cell proliferation is correlated with reduced levels of cell cycle protein CCND1, CDK4 and CDK6 (Fig. 1d). Similarly, SOX2 knockdown inhibits the proliferation of MDA-MB-231 cells (Fig. 1b right). We also performed colony forming assay to test whether SOX2 knockdown affects colony formation from single cells. Reduced levels of SOX2 affect the number and size of colonies. The number of colonies with a diameter greater than 0.5 mm formed from ZR7530 knockdown cells was significantly reduced in the SOX2-knockdwon (SOX2-Kd) group compared to scramble controls (*P* < 0.05) (Fig. 1c). Similarly, SOX2 knockdown in MDA-MB-231 cells also significantly reduced the number of colonies with diameters greater than 0.5 mm (*P* < 0.05). Together these data confirm that SOX2 is required for the proliferation of breast cancer cells.

**Fig. 2:**
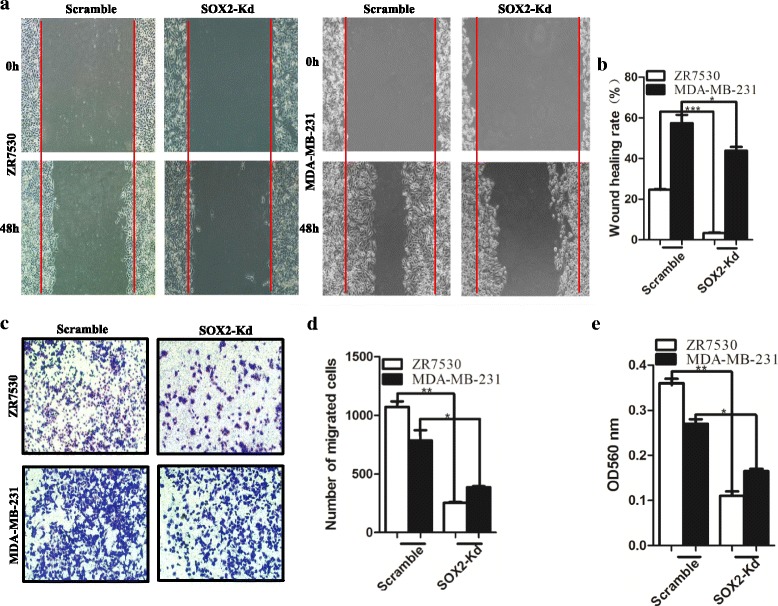
SOX2 is critical for the migration of breast cancer cells. **a** SOX2 knockdown suppresses the migration of ZR7530 and MDA-MB-231 cells in the wound-healing assay. **b** Quantification of cells migrating across the *red lines* in **a**. Data are represented as mean ± SD (*n* = 3 for each group, *p* < 0.05). **c** Down-regulation of SOX2 leads to reduced migration of breast cancer cells through the membrane in the transwell assay. **d** Quantification of cells that are present in the *lower side* of the transwell membrane. Data are represented as mean ± SD (*n* = 3 for each group, *p* < 0.05). **e** The intensity of dissolved violet staining is correlated with reduced number of cancer cells migrating through the membrane upon SOX2 knockdown. Cells stained with crystal violet at the *lower side* of the membrane were lysed and dissolved with acetic acid. The intensity was measured at an absorbance of 560 nm. * indicates *p* < 0.05, ** indicates *p* < 0.01, *** indicates *p* < 0.001 vs control, and the *error bars* indicate the mean ± standard deviation (SD)

High levels of SOX2 have been found in metastasized breast cancer nodules [42]. We next asked whether SOX2 is involved in cellular activities (e.g. cell migration) relevant to metastasis. SOX2 knockdown results in a decrease in the migration capability of ZR7530 and MDA-MB-231 cells (Fig. 2a). Wound-healing assay showed that the healing index was reduced from 24.75% (controls) to 3.33% for ZR7530 cells (*p* < 0.001), and from 57.39 to 43.8% for MDA-MB-231 cells (*p* < 0.05) (Fig. 2b). In addition, SOX2 knockdown inhibits the invasion of breast cancer cells in transwell assay. The number of cancer cells passing through the filter of the transwell are significantly reduced after SOX2 knockdown (Fig. 2c, d, *p* < 0.01 for ZR7530 and *p* < 0.05 for MDA-MB-231). The changed tendency of the OD value of eluted crystal violet was consistent with the number of migrated cells in these groups (*p* < 0.01 for ZR7530 and *p* < 0.05 for MDA-MB-231) (Fig. 2e). Together, these in vitro results demonstrate that high levels of SOX2 protein are critical for migration and invasion of breast cancer cells.

### SOX2 level changes are correlated with tumour development and metastasis in murine xenograft models

**Fig. 3:**
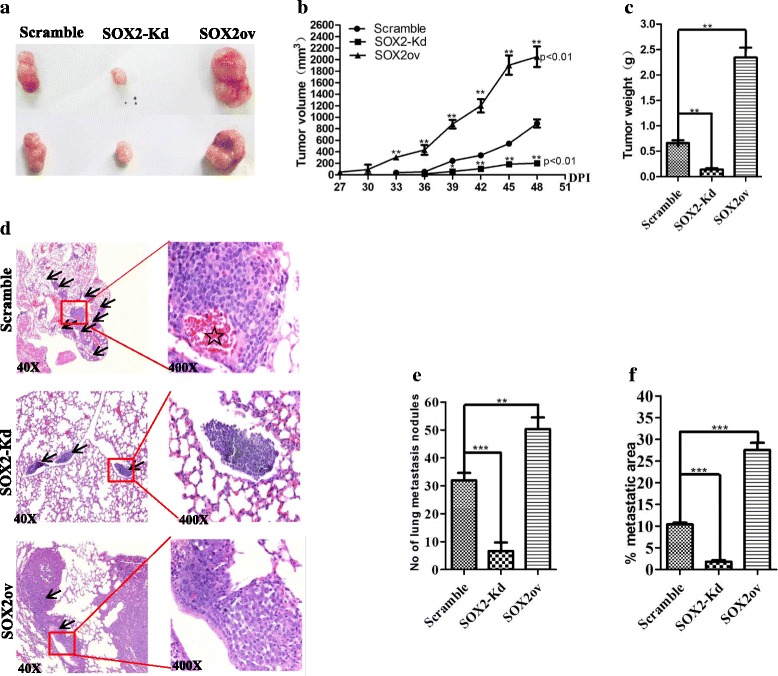
SOX2 knockdown reduces the growth and metastasis of breast cancer cells in xenograft models. **a** Representative xenografts formed by SOX2 knockdown, control and SOX2 overexpression breast cancer cells. Tumours were collected and examined 7 weeks after inoculation of ZR7530 cells. **b-c** SOX2 knockdown and SOX2 overexpression significantly reduces and increases the size and weight of xenografts, respectively (*n* = 5 per cohort, *p* < 0.01). **d**) Representative HE staining of metastatic foci in the lung tissues of BALB/c nude mice 8 weeks after injection with ZR7530 (SOX2 knockdown, control and SOX2 overexpression). Note the presence of blood vessels (*star*) in the tumour nodule of control group, *arrows point* to metastatic areas. **e**–**f** SOX2 knockdown significantly reduces the numbers of tumour nodule initiated by ZR7530 cells and it also reduces the ratio of metastatic tumour area/total lung area. However, SOX2 overexpression significantly increases the numbers of tumour nodule and the ratio of metastatic tumour area/total lung area. Data are represented as mean ± SD (*n* = 5 for each cohort, *p* < 0.001). * indicates *p* < 0.05, ** indicates *p* < 0.01, *** indicates *p* < 0.001 vs control, and the *error bars* indicate the mean ± standard deviation (SD)

We next used a xenograft model to test whether SOX2 knockdown reduces tumour growth in vivo. SOX2 knockdown ZR7530 and control cells were transplanted into immunocompromised nude mice. Significantly, SOX2 knockdown leads to 4.4 and 4.7 fold decreases in the volume and weight of tumour, respectively (Fig. 3a–c) (*n* = 5). In addition, SOX2 overexpression enables higher tumourigenesis capability of breast cancer cells. We transplanted SOX2-overexpressing ZR7530 cells into nude mice, and SOX2 overexpression results in a 2.3 fold increase in tumour volume and a 3.6 fold increase in tumour weight (Fig. 3a–c) (*n* = 5). These results suggest that the levels of SOX2 are correlated with increased growth capability of breast cancer cells in vivo.

Our in vitro results show that SOX2 knockdown reduces the migration of breast cancer cell lines. To test whether decreased levels of SOX2 also block metastasis capability in vivo, we performed tail vein injection of ZR7530 and studied tumour seeding and development in the lung. Upon SOX2 knockdown, the number of tumours seeded in the lung are significantly reduced (33 ± 2 Vs 7 ± 3) (*p* < 0.001) (*n* = 5) (Fig. 3d, e). In addition, SOX2 knockdown also reduces the ratio of tumour/total lung area (10.4% Vs 1.8%) (*p* < 0.001) (Fig. 3f). By contrast, ectopic SOX2 expression enables high metastastic capability of ZR7530 cells, leading to an increased number of seeding tumours (33 ± 2 Vs 51 ± 4) (*p* < 0.01) and enlarged metastatic foci in the lung (10.4% vs 27.5%) (*p* < 0.001) (Fig. 3d–f). Together, these in vivo findings support the important roles of SOX2 in promoting metastasis of breast cancer cells.

### SOX2 knockdown leads to decreased levels of miR-181a-5p and miR-30e-5p

**Fig. 4:**
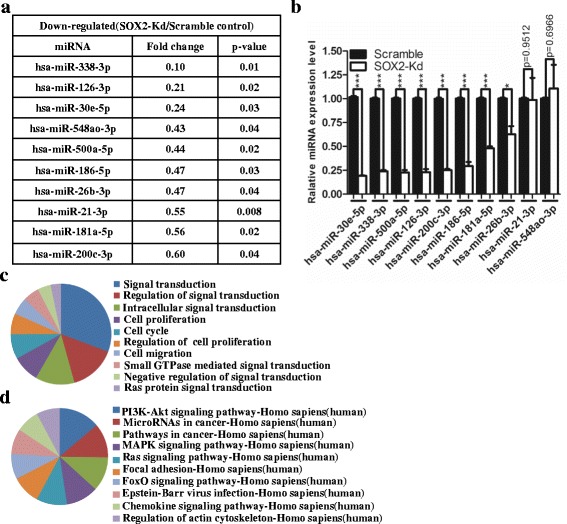
Decreased SOX2 levels are associated with reduced expression of miR-181a-5p and miR-30e-5p. **a** Unsupervised hierarchical cluster analysis of down-regulated miRNAs following SOX2 knockdown. **b** The decrease in the expression of microRNAs was validated with real-time PCR. Note the levels of miR-30e-5p are decreased most prominent following SOX2 knockdown. **c** Cellular activities affected by changes in the levels of microRNAs following SOX2 knockdown. **d** Cellular pathways affected by changes in the levels of microRNAs upon SOX2 knockdown. * indicates *p* < 0.05, ** indicates *p* < 0.01, *** indicates *p* < 0.001 vs control, and the *error bars* indicate the mean ± standard deviation (SD)

miRNAs have been implicated in the regulation of multiple steps in breast cancer progression [43–45]. To test whether SOX2 regulates proliferation and metastasis through miRNAs, we performed microRNA microarray analysis (miRCURY™ LNA Expression Array v.18.0, Exiqon). Total RNAs were isolated from scramble control and SOX2 knockdown ZR7530 cells. SOX2 knockdown significantly changed the levels of multiple miRNAs, among which 11 were up-regulated and 10 were significantly down-regulated (Fig. 4a and Additional file 1: Table S6, *p* < 0.05). We validated the expression levels with real-time PCR (Fig. 4b). Although the levels of miR-338-3p are significantly down-regulated in the array analysis, the levels of miR-30e-5p are most dramatically down-regulated upon SOX2 knockdown, which was validated by real-time PCR (Fig. 4b). Notably, the levels of miR-181a-5p which has previously been associated with breast cancer metastasis are also down-regulated (*p* < 0.001) (Fig. 4a, b) [46]. Down-regulation of miR-30e-5p and miR-181a-5p was also observed in MDA-MB-231 cells after SOX2 knockdown (data not shown).

### miR-181a-5p and miR-30e-5p co-regulate TUSC3 in breast cancer cells

**Fig. 5:**
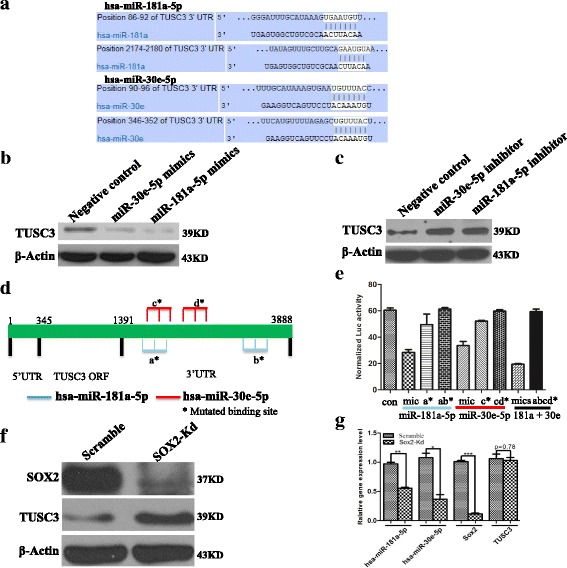
SOX2 modulates the expression of TUSC3 through miR-181a-5p and miR-30e-5p. **a** The sequence of the potential binding sites for miR-181a-5p and miR-30e-5p in the 3’-UTR of TUSC3. The sites are conserved in human and rodents. **b** The expression of TUSC3 is repressed by miR-181a-5p and miR-30e-5p mimics as measured by Western blot analysis. **c** The application of specific inhibitors against miR-181a-5p and miR-30e-5p increases the expression of TUSC3 in ZR7530 cells. **d** Schematic diagram of the binding sites for miR-181a-5p and miR-30e-5p in 3’-UTR of *TUSC* transcript. Mutated binding sites (*star*) were linked with Luciferase reporter (pMIR-Report-Luc). **e** Mutations of the potential binding sites for miR-181a-5p and miR-30e-5p lead to increased luciferase activities of *TUSC3* reporter. Co-transfection of negative mimics and pMIR-Report-Luc-TUSC3-3UTR-WT construct is used as control (Con). Mic indicates miR-181a-5p or miR-30e-5p mimics. Mics indicates mixture of miR-181a-5p and miR-30e-5p mimics, ab* indicates both of a* and b* potential bindings sites are mutated, cd* indicates both of c* and d* potential bindings sites are mutated, and abcd* indicates all four potential bindings sites are mutated. **f** SOX2 knockdown leads to increased protein levels of TUSC3. **g** In xenograft initiated by ZR7530 cells, SOX2 knockdown leads to reduced expression of miR-181a-5p and miR-30e-5p, but not the expression of TUSC3 mRNA. The transcript levels were measured by examining RNA expression in three individual tumour nodules in each group. * indicates *p* < 0.05, ** indicates *p* < 0.01, *** indicates *p* < 0.001 vs control, and the *error bars* indicate the mean ± standard deviation (SD)

Several miRNAs down-regulated upon SOX2 knockdown, have been implicated in the regulation of genes involved in metabolism, signalling pathway activation (Fig. 4c, d). Intriguingly, miR-181a-5p and miR-30e-5p share a common downstream target, TUSC3 as identified by the cytoscape program. TUSC3 is a putative tumor suppressor gene, and hypermethylation or loss of this gene has been found in cancers such as ovarian and breast cancer [47–49]. Intriguingly, two potential binding sites of miR-181a-5p and miR-30e-5p were identified at the 3’-UTR of TUSC3 (Fig. 5a). Consistently, transfection of miR-181a-5p or miR-30e-5p mimics reduces the protein levels of TUSC3 in ZR7530 cells (Fig. 5b). Conversely, inhibition of these two miRNAs with specific inhibitors leads to increased protein levels of TUSC3 (Fig. 5c). We then cloned the 3’-UTR of TUSC3 containing the potential miRNA binding sites into the luciferase reporter vectors and tested whether these two miRNAs regulate TUSC3 through the potential binding sites (Fig. 5d, Additional file 2: Figure S1). Interestingly, co-transfection of miR-181a-5p or miR-30e-5p mimics reduces the luciferase activities to 47 and 55.4% of control, respectively (Fig. 5e). More importantly, mutation of these potential binding sites leads to attenuated luciferase activities, suggesting that miR-181a-5p or miR-30e-5p controls TUSC3 expression through the binding sites. As expected, SOX2 knockdown leads to increased protein levels of TUSC3 protein (Fig. 5f). Moreover, in the xenografts of SOX2 knockdown group the transcript levels of SOX2 remain low and the level of miR-181a-5p or miR-30e-5p are also significantly lower than the control group (*p* < 0.05 for hsa-miR-30e-5p and *p* < 0.01 for hsa-miR-181a-5p), suggesting that SOX2 is required for maintaining the expression of two microRNAs in vivo. In addition, we did not see significant change of TUSC3 transcript levels, which is consistent with the finding that the two microRNA regulate translation but not transcription of TUSC3 (Fig. 5g). Taken together, these data indicate a regulatory axis where SOX2 modulates the levels of the tumor suppressor protein TUSC3 through miR-181a-5p and miR-30e-5p.

### TUSC3 regulates the proliferation, migration and invasion of breast cancer cells

**Fig. 6:**
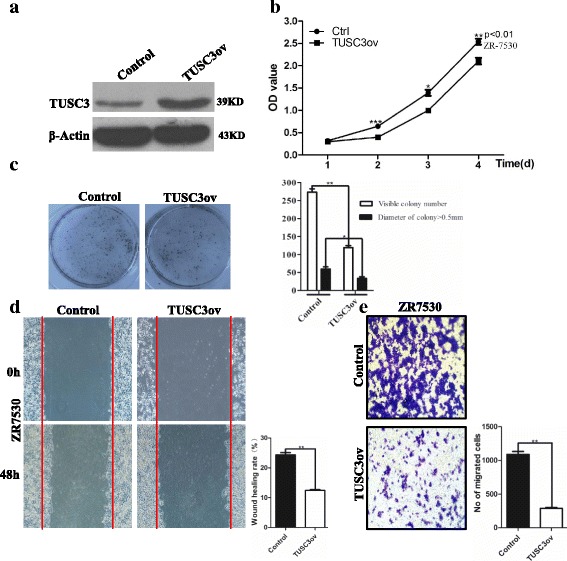
Increased levels of TUSC3 suppress the proliferation and migration of breast cancer cells. **a** Establishment of cell line (ZR7530) overexpressing TUSC3. **b** TUSC3 overexpression leads to reduced proliferation of ZR7530 cells. Data are represented as mean ± SD (*n* = 3 for each group, *p* < 0.01). **c** TUSC3 overexpression suppresses colony formation efficiency and reduces the size of individual clones in agar assay. Data are represented as mean ± SD (*n* = 3 for each group, *p* < 0.01). **d** Migration of cancer cells are inhibited by TUSC3 overexpression as revealed by wound healing assay (*p* < 0.01). **e** Overexpression of TUSC3 reduces the invasion capability of ZR7530 cells in the transwell assay (*p* < 0.01). The cell number present at the lower side of the membrane of the insert is counted and presented in the graph. * indicates *p* < 0.05, ** indicates *p* < 0.01, *** indicates *p* < 0.001 vs control, and the *error bars* indicate the mean ± standard deviation (SD)

SOX2 knockdown leads to decreased levels of miR-181a-5p and miR-30e-5p and increased levels of TUSC3. To test whether TUSC3 acts as a negative regulator of proliferation and migration of breast cancer cells, we performed TUSC3 gain-of-function study (Fig. 6a). TUSC3 overexpression leads to decreased proliferation of ZR7530 cells (*p* < 0.01) (Fig. 6b). In addition, TUSC3 overexpression significantly inhibits the colony formation efficiency (274 ± 6 Vs 119 ± 4 colonies/well) in 6-well plates (*p* < 0.01) and reduces the size of individual colonies (*p* < 0.05) (Fig. 6c), suggesting that TUSC3 acts as a negative regulator of proliferation of breast cancer cells.

We next asked whether increased levels of TUSC3 are also able to reduce the migration of breast cancer cells. We first used a wound-healing assay to address the issue and observed that TUSC3 overexpression significantly reduced the healing index of ZR7530 cells as compared to control (24.3% Vs 12.4%) (*p* < 0.01) (Fig. 6d). We then used the transwell assay to further test the effect of TUSC3 overexpression on cell invasion. Similar to SOX2 knockdown, TUSC3 overexpression dramatically decreased the invasion capability of ZR7530 (*p* < 0.01) (Fig. 6e). Collectively, these results demonstrated that high levels of TUSC3 protein reduce the invasive potential of breast cancer cells.

### Increased SOX2 protein levels correlate with poor survival in breast cancer patients

**Fig. 7:**
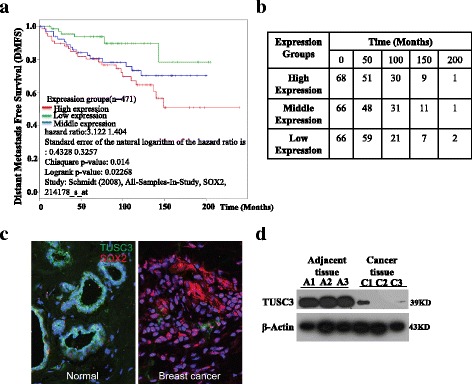
Expression of SOX2 and TUSC3 in human breast cancer samples. **a** High levels of SOX2 are correlated with poor distant metastasis-free survival. **b** Patient survival time and its correlation with SOX2 expression levels. **c** High levels of SOX2 protein are present in representative breast cancer sample which has low levels of TUSC3 (*n* = 9 pairs). Note the normal breast tissue expresses relatively high levels of TUSC3. **d** Breast cancer biopsies express very low levels of TUSC3 protein as compared to adjacent normal tissues. Biopsies were taken from three patients

Previous studies revealed that approximately 16% of breast cancer patients exhibit SOX2-positive expression in clinical samples, and SOX2 is expressed in the early stage of breast tumours [17]. However, other studies revealed that SOX2 expression was associated with subtypes and tumour grades of breast cancer [5, 50]. To analyse whether Sox2 expression level (by probe 214178_s_at) correlates with distant metastasis free survival in breast cancer patients, we used the Kaplan-Meier analysis from IBM SPSS software to analyse the relationship between SOX2 protein levels and 5-year survival rates in 471 patients, and the patients data used for analysis derives from previous study [51]. As shown in Fig. 7a, SOX2 expression level significantly converts poor prognosis (Chisquare *p*-value = 0.014, Logrank *p*-value = 0.02268), and the distant metastasis-free survival of breast cancer patients expressing high, middle and low levels of SOX2 protein were 50.4, 70.5 and 78.6%, suggesting that high levels of SOX2 are correlated with poor prognosis in breast cancer patients. Further analysis revealed that patients with high and middle levels of SOX2 have a significant worse survival rate than those with low levels (Fig. 7a, b) (*p* < 0.05). However, the survival difference between the middle and low level groups is not significant (p > 0.05). Moreover, we found that SOX2 levels are inversely correlated with those of TUSC3. We examined 12 pairs of normal and breast cancer biopsies, and found that 9 breast cancer biopsies have increased expression of SOX2 proteins concomitant with low levels of TUSC3 (Fig. 7c). In keeping with this line, high levels of TUSC3 protein were detected in adjacent normal tissue samples but were very low in cancer biopsies (Fig. 7d).

## Discussion

High levels of SOX2 proteins have been linked to breast cancer metastasis. However, the underlying mechanisms remain largely unknown. Here, we use in vitro and in vivo models to demonstrate that SOX2 is required for multiple steps in metastatic processes. We showed that SOX2 regulates the proliferation, migration and seeding of breast cancer cells. More importantly, we have identified a miRNA-mediated regulatory axis downstream of SOX2 to control these processes. Specifically, we found that SOX2 regulates the levels of miR-181a-5p and miR-30e-5p which in turn modulate the levels of the tumor suppressor TUSC3. Consistently, overexpression of TUSC3 reduces the proliferation and migration of SOX2-high breast cancer cells.

SOX2 is a key transcription factor important for the self-renewal of stem cells in multiple tissues including the trachea and brain [6]. We and others have shown that SOX2 overexpression promotes the proliferation of stem cells in the esophagus and lung, leading to malignancy [15, 33], suggesting that SOX2 acts as an oncogene. In this study, we found that SOX2 is also required for the proliferation of breast cancer cells. SOX2 knockdown leads to decreased levels of cell cycle proteins including CCND1, CDK4 and CDK6. Interestingly, SOX2 knockdown also leads to reduced levels of multiple microRNAs. We found that the levels of miR-30e-5p (previously named as miR-30e) are most dramatically down-regulated upon SOX2 knockdown, which was validated by real-time PCR. Interestingly, we also found that miR-181a-5p (previously named as miR-181a) which has previously been linked to breast cancer development is also dramatically downregulated. More importantly, further analysis using the cytoscape program we found that miR-30e-5p together with miR-181a-5p co-regulate a common downstream target, TUSC3. Both miR-30e-5p and miR-181a-5p have been implicated in the regulation of cancer cell growth and migration, invasion and metastasis [52–54]. miR-181a-5p is a member of the miR-181 s family which includes three other highly conserved miRNAs, miR-181b, c, and d [55]. Previous studies have shown increased levels of miR-181a-5p in multiple types of cancer including ovarian cancer and gastric cancer [56, 57]. The levels of miR-181a-5p in breast cancer were recently assessed. While Li et al. demonstrated that miR-181a-5p is down-regulated in aggressive human breast and colon cancers [58], a recent study showed that the levels of miR-181a-5p are high in breast cancer samples [59]. By contrast, we have just begun to elicit the role of miR-30e-5p in tumour development. Tissue microarray analysis suggests that high levels of miR-30e-5p in primary tumours are associated with a favourable prognosis in breast cancer [60]. In our study, we observed increased levels of miR-181a-5p and miR-30e-5p in breast cancer biopsies as compared to normal mammary gland tissue. We found that SOX2 knockdown leads to decreased levels of miR-181a-5p and miR-30e-5p accompanied by decreased cell proliferation, suggesting that these two microRNAs are required for the proliferation of breast cancer cells.

Consistently, we identified that miR-181a-5p and miR-30e-5p target the tumor suppressor TUSC3. Overexpression of these two miRNAs reduces the protein levels of TUSC3. Moreover, the luciferase assay showed that miR-181a-5p and miR-30e-5p regulate the 3’-UTR of TUSC3 transcripts. TUSC3 is a putative tumour suppressor often lost in epithelial cancers e.g. ovarian cancer and head and neck squamous cell carcinomas [47, 61]. Promoter methylation leading to silencing of *TUSC3* gene was considered as a poor prognostic factor in ovarian cancer [48]. Here, we show decreased but not complete loss of TUSC3 protein in breast cancers, suggesting that promoter methylation is unlikely a significant contributor to disease progression. We found that the protein levels of TUSC3 are decreased in breast tumour tissues as compared to surrounding normal tissues. In addition, overexpression of TUSC3 decreases the proliferation and migration capabilities of breast cancer cells. These findings are consistent with the tumor suppressor function of TUSC3 [62]. TUSC3 is an important component of the oligosaccharyltransferase (OST) complex that catalyzes N-linked glycosylation of proteins in the endoplasmic reticulum (ER) [63, 64]. Loss of TUSC3 in mammalian cells leads to accumulation of unfolded or misfolded proteins in the ER, resulting in ER stress. In ovarian cancer cells loss of TUSC3 induces ER stress and morphological alterations in the ER. Interestingly, ovarian cancer cells lacking TUSC3 seem able to alleviate the massive ER stress response through the PERK-mediated adaptive pathway [65], which is consistent with the finding that accumulated unfolded proteins in cancer cells induce the unfolded protein response (UPR), facilitating cancer cells to adapt to ER stress [66]. miR-181a-5p overexpression has been shown to promote ER stress and leads to myogenic differentiation in C2C12 cells [67]. Along these lines our study indicates that high levels of TUSC3 expression may induce a cellular response of breast cancer cells, resulting in reduced cell proliferation and migration.

Previous studies revealed that SOX2 protein levels are correlated with the overall survival of patients with multiple types of cancer including esophageal [33] and lung cancer [24]. Moreover, SOX2 protein has been assessed as a diagnostic marker and therapeutic target for multiple cancer treatments [68–70]. Our results suggest that high levels of SOX2 protein are associated with poor prognosis of breast cancer.

## Conclusions

**Fig. 8:**
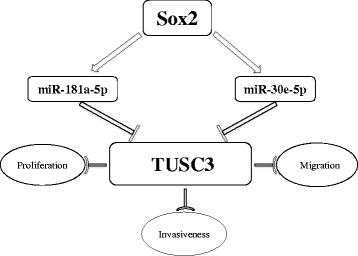
Working Model: SOX2 regulates the proliferation, migration and invasiveness of breast cancer cells through miR-181a-5p and miR-30e-5p which modulate TUSC3 protein levels

In summary, we showed in this study that SOX2 protein is critical for the proliferation, migration and metastasis of breast cancer. We identified that SOX2 exerts these functions through miR-181a-5p and miR-30e-5p which share a common downstream target, the putative tumour suppressor TUSC3 (Fig. 8). Knockdown SOX2 leads to decreased levels of miR-181a-5p and miR-30e-5p accompanied by increased levels of TUSC3. Consistently, overexpression of TUSC3 suppresses the proliferation and migration of breast cancer cells. Together these findings support a regulatory axis orchestrated by high levels of SOX2, and this axis is critical for breast cancer development. In future studies it will be interesting to explore potential avenues to target this SOX2/microRNA/TUSC3 axis for therapeutic gains.

## Additional files

Additional file 1: Table S1. Primers used for generating TUSC3 overexpression construct. **Table S2.** Stem-loop reverse transcription primers used for detecting miRNAs expression. **Table S3.** qPCR Primers used for detecting miRNAs expression. **Table S4.** Primers used for generating Luciferase reporters. **Table S5.** Sequences of miRNA mimics and inhibitors. **Table S6.** microRNAs that were significantly up-regulated in ZR7530 following *SOX2* knockdown as detected by microarray analysis. (DOC 83 kb)
Additional file 2: Figure S1. Construction of Luciferase reporters. (DOC 158 kb)

## Abbreviations

CCK-8: Cell Counting Kit 8
ER: Endoplasmic reticulum
ESCC: Esophageal squamous cell carcinomas
GO: Gene Ontology
HE: Haematoxylin and eosin
miRNAs: MicroRNAs
OST: Oligosaccharyltransferase
SAM: Significance Analysis of Microarrays
SD: Standard deviation
SOX: Sex determining region Y (SRY)-like box
SOX2-Kd: SOX2-knockdwon
TUSC3: Tumor Suppressor Candidate 3
UPR: Unfolded protein response

## References

[1] Cardoso F, Harbeck N, Fallowfield L, Kyriakides S, Senkus E (2012). Locally recurrent or metastatic breast cancer: ESMO Clinical Practice Guidelines for diagnosis, treatment and follow-up. Ann Oncol.

[2] Teng YH, Tan WJ, Thike AA, Cheok PY, Tse GM, Wong NS, Yip GW, Bay BH, Tan PH (2011). Mutations in the epidermal growth factor receptor (EGFR) gene in triple negative breast cancer: possible implications for targeted therapy. Breast Cancer Res.

[3] Bose R, Kavuri SM, Searleman AC, Shen W, Shen D, Koboldt DC, Monsey J, Goel N, Aronson AB, Li S (2013). Activating HER2 mutations in HER2 gene amplification negative breast cancer. Cancer Discov.

[4] Zabransky DJ, Yankaskas CL, Cochran RL, Wong HY, Croessmann S, Chu D, Kavuri SM, Red Brewer M, Rosen DM, Dalton WB (2015). HER2 missense mutations have distinct effects on oncogenic signaling and migration. Proc Natl Acad Sci U S A.

[5] Li X, Xu Y, Chen Y, Chen S, Jia X, Sun T, Liu Y, Xiang R, Li N (2013). SOX2 promotes tumor metastasis by stimulating epithelial-to-mesenchymal transition via regulation of WNT/beta-catenin signal network. Cancer Lett.

[6] Que J, Luo X, Schwartz RJ, Hogan BL (2009). Multiple roles for Sox2 in the developing and adult mouse trachea. Development.

[7] Li XL, Eishi Y, Bai YQ, Sakai H, Akiyama Y, Tani M, Takizawa T, Koike M, Yuasa Y (2004). Expression of the SRY-related HMG box protein SOX2 in human gastric carcinoma. Int J Oncol.

[8] Takahashi K, Yamanaka S (2006). Induction of pluripotent stem cells from mouse embryonic and adult fibroblast cultures by defined factors. Cell.

[9] Takahashi K, Tanabe K, Ohnuki M, Narita M, Ichisaka T, Tomoda K, Yamanaka S (2007). Induction of pluripotent stem cells from adult human fibroblasts by defined factors. Cell.

[10] Fong H, Hohenstein KA, Donovan PJ (2008). Regulation of self-renewal and pluripotency by Sox2 in human embryonic stem cells. Stem Cells.

[11] Wakamatsu Y, Endo Y, Osumi N, Weston JA (2004). Multiple roles of Sox2, an HMG-box transcription factor in avian neural crest development. Dev Dyn.

[12] Schepers GE, Teasdale RD, Koopman P (2002). Twenty pairs of sox: extent, homology, and nomenclature of the mouse and human sox transcription factor gene families. Dev Cell.

[13] Bass AJ, Watanabe H, Mermel CH, Yu S, Perner S, Verhaak RG, Kim SY, Wardwell L, Tamayo P, Gat-Viks I (2009). SOX2 is an amplified lineage-survival oncogene in lung and esophageal squamous cell carcinomas. Nat Genet.

[14] Gen Y, Yasui K, Zen Y, Zen K, Dohi O, Endo M, Tsuji K, Wakabayashi N, Itoh Y, Naito Y (2010). SOX2 identified as a target gene for the amplification at 3q26 that is frequently detected in esophageal squamous cell carcinoma. Cancer Genet Cytogenet.

[15] Lu Y, Futtner C, Rock JR, Xu X, Whitworth W, Hogan BL, Onaitis MW (2010). Evidence that SOX2 overexpression is oncogenic in the lung. Plos One.

[16] Santini R, Pietrobono S, Pandolfi S, Montagnani V, D’Amico M, Penachioni JY, Vinci MC, Borgognoni L, Stecca B (2014). SOX2 regulates self-renewal and tumorigenicity of human melanoma-initiating cells. Oncogene.

[17] Leis O, Eguiara A, Lopez-Arribillaga E, Alberdi MJ, Hernandez-Garcia S, Elorriaga K, Pandiella A, Rezola R, Martin AG (2012). Sox2 expression in breast tumours and activation in breast cancer stem cells. Oncogene.

[18] Basu-Roy U, Seo E, Ramanathapuram L, Rapp TB, Perry JA, Orkin SH, Mansukhani A, Basilico C (2012). Sox2 maintains self renewal of tumor-initiating cells in osteosarcomas. Oncogene.

[19] Neumann J, Bahr F, Horst D, Kriegl L, Engel J, Luque RM, Gerhard M, Kirchner T, Jung A (2011). SOX2 expression correlates with lymph-node metastases and distant spread in right-sided colon cancer. BMC Cancer.

[20] Xiang R, Liao D, Cheng T, Zhou H, Shi Q, Chuang TS, Markowitz D, Reisfeld RA, Luo Y (2011). Downregulation of transcription factor SOX2 in cancer stem cells suppresses growth and metastasis of lung cancer. Br J Cancer.

[21] Sun C, Sun L, Li Y, Kang X, Zhang S, Liu Y (2013). Sox2 expression predicts poor survival of hepatocellular carcinoma patients and it promotes liver cancer cell invasion by activating Slug. Med Oncol.

[22] Hussenet T, Dali S, Exinger J, Monga B, Jost B, Dembele D, Martinet N, Thibault C, Huelsken J, Brambilla E, du Manoir S (2010). SOX2 is an oncogene activated by recurrent 3q26.3 amplifications in human lung squamous cell carcinomas. Plos One.

[23] Mccaughan F, Pole JC, Bankier AT, Konfortov BA, Carroll B, Falzon M, Rabbitts TH, George PJ, Dear PH, Rabbitts PH (2010). Progressive 3q amplification consistently targets SOX2 in preinvasive squamous lung cancer. Am J Respir Crit Care Med.

[24] Wilbertz T, Wagner P, Petersen K, Stiedl AC, Scheble VJ, Maier S, Reischl M, Mikut R, Altorki NK, Moch H (2011). SOX2 gene amplification and protein overexpression are associated with better outcome in squamous cell lung cancer. Mod Pathol.

[25] Rudin CM, Durinck S, Stawiski EW, Poirier JT, Modrusan Z, Shames DS, Bergbower EA, Guan Y, Shin J, Guillory J (2012). Comprehensive genomic analysis identifies SOX2 as a frequently amplified gene in small-cell lung cancer. Nat Genet.

[26] Belotte J, Fletcher NM, Alexis M, Morris RT, Munkarah AR, Diamond MP, Saed GM (2015). Sox2 gene amplification significantly impacts overall survival in serous epithelial ovarian cancer. Reprod Sci.

[27] Piva M, Domenici G, Iriondo O, Rabano M, Simoes BM, Comaills V, Barredo I, Lopez-Ruiz JA, Zabalza I, Kypta R, Vivanco M (2014). Sox2 promotes tamoxifen resistance in breast cancer cells. EMBO Mol Med.

[28] Herranz H, Cohen SM (2010). MicroRNAs and gene regulatory networks: managing the impact of noise in biological systems. Genes Dev.

[29] Mulrane L, Mcgee SF, Gallagher WM, O’Connor DP (2013). miRNA dysregulation in breast cancer. Cancer Res.

[30] Zhang Y, Yang P, Sun T, Li D, Xu X, Rui Y, Li C, Chong M, Ibrahim T, Mercatali L (2013). miR-126 and miR-126* repress recruitment of mesenchymal stem cells and inflammatory monocytes to inhibit breast cancer metastasis. Nat Cell Biol.

[31] Hu J, Sun T, Wang H, Chen Z, Wang S, Yuan L, Liu T, Li HR, Wang P, Feng Y (2016). MiR-215 Is Induced Post-transcriptionally via HIF-Drosha Complex and Mediates Glioma-Initiating Cell Adaptation to Hypoxia by Targeting KDM1B. Cancer Cell.

[32] Seviour EG, Sehgal V, Lu Y, Luo Z, Moss T, Zhang F, Hill SM, Liu W, Maiti SN, Cooper L (2016). Functional proteomics identifies miRNAs to target a p27/Myc/phospho-Rb signature in breast and ovarian cancer. Oncogene.

[33] Liu K, Jiang M, Lu Y, Chen H, Sun J, Wu S, Ku WY, Nakagawa H, Kita Y, Natsugoe S (2013). Sox2 cooperates with inflammation-mediated Stat3 activation in the malignant transformation of foregut basal progenitor cells. Cell Stem Cell.

[34] Jiang M, Ku WY, Zhou Z, Dellon ES, Falk GW, Nakagawa H, Wang ML, Liu K, Wang J, Katzka DA (2015). BMP-driven NRF2 activation in esophageal basal cell differentiation and eosinophilic esophagitis. J Clin Invest.

[35] Ikezoe T, Gery S, Yin D, O’Kelly J, Binderup L, Lemp N, Taguchi H, Koeffler HP (2005). CCAAT/enhancer-binding protein delta: a molecular target of 1,25-dihydroxyvitamin D3 in androgen-responsive prostate cancer LNCaP cells. Cancer Res.

[36] Chang PH, Hwang-Verslues WW, Chang YC, Chen CC, Hsiao M, Jeng YM, Chang KJ, Lee EY, Shew JY, Lee WH (2012). Activation of Robo1 signaling of breast cancer cells by Slit2 from stromal fibroblast restrains tumorigenesis via blocking PI3K/Akt/beta-catenin pathway. Cancer Res.

[37] Kliment CR, Englert JM, Gochuico BR, Yu G, Kaminski N, Rosas I, Oury TD (2009). Oxidative stress alters syndecan-1 distribution in lungs with pulmonary fibrosis. J Biol Chem.

[38] Basile KJ, Abel EV, Dadpey N, Hartsough EJ, Fortina P, Aplin AE (2013). In vivo MAPK reporting reveals the heterogeneity in tumoral selection of resistance to RAF inhibitors. Cancer Res.

[39] Veitonmaki N, Hansson M, Zhan F, Sundberg A, Lofstedt T, Ljungars A, Li ZC, Martinsson-Niskanen T, Zeng M, Yang Y (2013). A human ICAM-1 antibody isolated by a function-first approach has potent macrophage-dependent antimyeloma activity in vivo. Cancer Cell.

[40] Cooke VG, Lebleu VS, Keskin D, Khan Z, O’Connell JT, Teng Y, Duncan MB, Xie L, Maeda G, Vong S (2012). Pericyte depletion results in hypoxia-associated epithelial-to-mesenchymal transition and metastasis mediated by met signaling pathway. Cancer Cell.

[41] Lebleu VS, O’Connell JT, Gonzalez Herrera KN, Wikman H, Pantel K, Haigis MC, de Carvalho FM, Damascena A, Domingos Chinen LT, Rocha RM (2014). PGC-1alpha mediates mitochondrial biogenesis and oxidative phosphorylation in cancer cells to promote metastasis. Nat Cell Biol.

[42] Huang YH, Luo MH, Ni YB, Tsang JY, Chan SK, Lui PC, Yu AM, Tan PH, Tse GM (2014). Increased SOX2 expression in less differentiated breast carcinomas and their lymph node metastases. Histopathology.

[43] Liu H (2012). MicroRNAs in breast cancer initiation and progression. Cell Mol Life Sci.

[44] Bockhorn J, Yee K, Chang YF, Prat A, Huo D, Nwachukwu C, Dalton R, Huang S, Swanson KE, Perou CM (2013). MicroRNA-30c targets cytoskeleton genes involved in breast cancer cell invasion. Breast Cancer Res Treat.

[45] Bockhorn J, Dalton R, Nwachukwu C, Huang S, Prat A, Yee K, Chang YF, Huo D, Wen Y, Swanson KE (2013). MicroRNA-30c inhibits human breast tumour chemotherapy resistance by regulating TWF1 and IL-11. Nat Commun.

[46] Neel JC, Lebrun JJ (2013). Activin and TGFbeta regulate expression of the microRNA-181 family to promote cell migration and invasion in breast cancer cells. Cell Signal.

[47] Vanhara P, Horak P, Pils D, Anees M, Petz M, Gregor W, Zeillinger R, Krainer M (2013). Loss of the oligosaccharyl transferase subunit TUSC3 promotes proliferation and migration of ovarian cancer cells. Int J Oncol.

[48] Pils D, Horak P, Vanhara P, Anees M, Petz M, Alfanz A, Gugerell A, Wittinger M, Gleiss A, Auner V (2013). Methylation status of TUSC3 is a prognostic factor in ovarian cancer. Cancer.

[49] Conway K, Edmiston SN, Tse CK, Bryant C, Kuan PF, Hair BY, Parrish EA, May R, Swift-Scanlan T (2015). Racial variation in breast tumor promoter methylation in the Carolina Breast Cancer Study. Cancer Epidemiol Biomarkers Prev.

[50] Chen Y, Shi L, Zhang L, Li R, Liang J, Yu W, Sun L, Yang X, Wang Y, Zhang Y, Shang Y (2008). The molecular mechanism governing the oncogenic potential of SOX2 in breast cancer. J Biol Chem.

[51] Schmidt ME, Steindorf K, Mutschelknauss E, Slanger T, Kropp S, Obi N, Flesch-Janys D, Chang-Claude J (2008). Physical activity and postmenopausal breast cancer: effect modification by breast cancer subtypes and effective periods in life. Cancer Epidemiol Biomarkers Prev.

[52] Kwak SY, Kim BY, Ahn HJ, Yoo JO, Kim J, Bae IH, Han YH (2015). Ionizing radiation-inducible miR-30e promotes glioma cell invasion through EGFR stabilization by directly targeting CBL-B. FEBS J.

[53] Ji D, Chen Z, Li M, Zhan T, Yao Y, Zhang Z, Xi J, Yan L, Gu J (2014). MicroRNA-181a promotes tumor growth and liver metastasis in colorectal cancer by targeting the tumor suppressor WIF-1. Mol Cancer.

[54] Taylor MA, Sossey-Alaoui K, Thompson CL, Danielpour D, Schiemann WP (2013). TGF-beta upregulates miR-181a expression to promote breast cancer metastasis. J Clin Invest.

[55] Ji J, Yamashita T, Budhu A, Forgues M, Jia HL, Li C, Deng C, Wauthier E, Reid LM, Ye QH (2009). Identification of microRNA-181 by genome-wide screening as a critical player in EpCAM-positive hepatic cancer stem cells. Hepatology.

[56] Parikh A, Lee C, Joseph P, Marchini S, Baccarini A, Kolev V, Romualdi C, Fruscio R, Shah H, Wang F (2014). microRNA-181a has a critical role in ovarian cancer progression through the regulation of the epithelial-mesenchymal transition. Nat Commun.

[57] Zhang X, Nie Y, Du Y, Cao J, Shen B, Li Y (2012). MicroRNA-181a promotes gastric cancer by negatively regulating tumor suppressor KLF6. Tumour Biol.

[58] Li Y, Kuscu C, Banach A, Zhang Q, Pulkoski-Gross A, Kim D, Liu J, Roth E, Li E, Shroyer KR (2015). miR-181a-5p Inhibits Cancer Cell Migration and Angiogenesis via Downregulation of Matrix Metalloproteinase-14. Cancer Res.

[59] Ouyang M, Li Y, Ye S, Ma J, Lu L, Lv W, Chang G, Li X, Li Q, Wang S, Wang W (2014). MicroRNA profiling implies new markers of chemoresistance of triple-negative breast cancer. Plos One.

[60] D’Aiuto F, Callari M, Dugo M, Merlino G, Musella V, Miodini P, Paolini B, Cappelletti V, Daidone MG (2015). miR-30e* is an independent subtype-specific prognostic marker in breast cancer. Br J Cancer.

[61] Guervos MA, Marcos CA, Hermsen M, Nuno AS, Suarez C, Llorente JL (2007). Deletions of N33, STK11 and TP53 are involved in the development of lymph node metastasis in larynx and pharynx carcinomas. Cell Oncol.

[62] Horak P, Tomasich E, Vanhara P, Kratochvilova K, Anees M, Marhold M, Lemberger CE, Gerschpacher M, Horvat R, Sibilia M (2014). TUSC3 loss alters the ER stress response and accelerates prostate cancer growth in vivo. Sci Rep.

[63] Mohorko E, Glockshuber R, Aebi M (2011). Oligosaccharyltransferase: the central enzyme of N-linked protein glycosylation. J Inherit Metab Dis.

[64] Mohorko E, Owen RL, Malojcic G, Brozzo MS, Aebi M, Glockshuber R (2014). Structural basis of substrate specificity of human oligosaccharyl transferase subunit N33/Tusc3 and its role in regulating protein N-glycosylation. Structure.

[65] Kratochvilova K, Horak P, Esner M, Soucek K, Pils D, Anees M, Tomasich E, Drafi F, Jurtikova V, Hampl A (2015). Tumor suppressor candidate 3 (TUSC3) prevents the epithelial-to-mesenchymal transition and inhibits tumor growth by modulating the endoplasmic reticulum stress response in ovarian cancer cells. Int J Cancer.

[66] Hetz C, Martinon F, Rodriguez D, Glimcher LH (2011). The unfolded protein response: integrating stress signals through the stress sensor IRE1alpha. Physiol Rev.

[67] Wei Y, Tao X, Xu H, Chen Y, Zhu L, Tang G, Li M, Jiang A, Shuai S, Ma J (2016). Role of miR-181a-5p and endoplasmic reticulum stress in the regulation of myogenic differentiation. Gene.

[68] Li X, Chen S, Sun T, Xu Y, Chen Y, Liu Y, Xiang R, Li N (2014). The transcriptional regulation of SOX2 on FOXA1 gene and its application in diagnosis of human breast and lung cancers. Clin Lab.

[69] Polakova I, Duskova M, Smahel M (2014). Antitumor DNA vaccination against the Sox2 transcription factor. Int J Oncol.

[70] Finicelli M, Benedetti G, Squillaro T, Pistilli B, Marcellusi A, Mariani P, Santinelli A, Latini L, Galderisi U, Giordano A (2014). Expression of stemness genes in primary breast cancer tissues: the role of SOX2 as a prognostic marker for detection of early recurrence. Oncotarget.

