# Supplementary material for: SOX2 regulates multiple malignant processes of breast cancer development through the SOX2/miR-181a-5p, miR-30e-5p/TUSC3 axis
Source: Mol Cancer. 2017 Mar 14;16:62. doi: 10.1186/s12943-017-0632-9 (PMC5348847; doi:10.1186/s12943-017-0632-9)
Supplement: Additional file 1: Table S1. — Primers used for generating TUSC3 overexpression construct. Table S2. Stem-loop reverse transcription primers used for detecting miRNAs expression. Table S3. qPCR Primers used for detecting miRNAs expression. Table S4. Primers used for generating Luciferase reporters. Table S5. Sequences of miRNA mimics and inhibitors. Table S6. microRNAs that were significantly up-regulated in ZR7530 following SOX2 knockdown as detected by microarray analysis. (DOC 83 kb) [file 12943_2017_632_MOESM1_ESM.doc]

**Additional file 1: Table S1. Primers used for generating TUSC3 overexpression construct.**

| Plasmid | Primer sequence(5’-3’) |
| --- | --- |
| pCDH-CMV-TUSC3-IRES-GFP-EF1-Puro | Forward:5’-CGGAATTCGCCACCATGGGGGCCCGGGGCGCT-3’  Reverse:5’-CGGGATCCTCACTCAAAGTCCAGATC-3’ |

**All listed primers were designed using sequences from Gene Bank. pCDH-CMV-TUSC3-IRES-GFP-EF1-Puro was generated by inserting PCR products digested with EcoRI and BamHI into linearized pCDH-CMV-Sox2-IRES-GFP-EF1-Puro.**

**Additional file 1: Table S2. Stem-loop reverse transcription primers used for detecting**

**miRNAs expression**

| Genes | Primer sequence(5’-3’) |
| --- | --- |
| U6 | 5’-CGCTTCACGAATTTGCGTGTCAT-3’ |
| hsa-miR-30e-5p | 5’-GTCGTATCCAGTGCGTGTCGTGGAGTCGGCAATTGCACTGGATACGACC  TTCCA-3’ |
| hsa-miR-338-3p | 5’-GTCGTATCCAGTGCGTGTCGTGGAGTCGGCAATTGCACTGGATACGACT  CAACAAA-3’ |
| hsa-miR-500a-5p | 5’-GTCGTATCCAGTGCGTGTCGTGGAGTCGGCAATTGCACTGGATACGACT  CTCAC-3’ |
| hsa-miR-126-3p | 5’-GTCGTATCCAGTGCGTGTCGTGGAGTCGGCAATTGCACTGGATACGACC  GCATT-3’ |
| hsa-miR-200c-3p | 5’-GTCGTATCCAGTGCGTGTCGTGGAGTCGGCAATTGCACTGGATACGACTC  CATC-3’ |
| hsa-miR-186-5p | 5’-GTCGTATCCAGTGCGTGTCGTGGAGTCGGCAATTGCACTGGATACGACAG  CCCAA-3’ |
| hsa-miR-181a-5p | 5’-GTCGTATCCAGTGCGTGTCGTGGAGTCGGCAATTGCACTGGATACGACAC  TCAC-3’ |
| hsa-miR-26b-3p | 5’-GTCGTATCCAGTGCGTGTCGTGGAGTCGGCAATTGCACTGGATACGACGA  GCCA-3’ |
| hsa-miR-21-3p | 5’-GTCGTATCCAGTGCGTGTCGTGGAGTCGGCAATTGCACTGGATACGACAC  AGCC-3’ |
| hsa-miR-548ao-3p | 5’-GTCGTATCCAGTGCGTGTCGTGGAGTCGGCAATTGCACTGGATACGACT  GCAAA-3’ |

**Additional file 1: Table S3. qPCR Primers used for detecting miRNAs expression**

| Genes | Primer sequence(5’-3’) | Annealing temperature  (℃) | Length of targets |
| --- | --- | --- | --- |
| U6 | Forward:5’-GCTTCGGCAGCACATATACTAAAAT-3’  Reverse:5’-CGCTTCACGAATTTGCGTGTCAT-3’ | 60 | 89 |
| hsa-miR-30e-5p | Forward:5’-GGGTGTAAACATCCTTGAC-3’  Reverse:5’-TGCGTGTCGTGGAGTC-3’ | 60 | 62 |
| hsa-miR-338-3p | Forward:5’-GGGTCCAGCATCAGTGA-3’  Reverse:5’-CAGTGCGTGTCGTGGA-3’ | 60 | 66 |
| hsa-miR-500a-5p | Forward: 5’-GGCTAATCCTTGCTACCTGG-3’  Reverse: 5’-GTGCGTGTCGTGGAGTCG-3’ | 60 | 64 |
| hsa-miR-126-3p | Forward: 5’-GGGGTCGTACCGTGAGTAAT-3’  Reverse: 5’-GTGCGTGTCGTGGAGTCG-3’ | 60 | 64 |
| hsa-miR-200c-3p | Forward:5’-TGGGTAATACTGCCGGGTAAT-3’  Reverse:5’-GTGCGTGTCGTGGAGTCG-3’ | 60 | 65 |
| hsa-miR-186-5p | Forward: 5’-TCCCCCAAAGAATTCTCCTT-3’  Reverse: 5’-GTGCGTGTCGTGGAGTCG-3’ | 60 | 65 |
| hsa-miR-181a-5p | Forward:5’-GGGAACATTCAACGCTGTCG-3’  Reverse:5’-GTGCGTGTCGTGGAGTCG-3’ | 60 | 64 |
| hsa-miR-26b-3p | Forward: 5’-GGGGTCCTGTTCTCCATTACT-3’  Reverse: 5’-GTGCGTGTCGTGGAGTCG-3’ | 60 | 64 |
| hsa-miR-21-3p | Forward: 5’-GGCAACAGCAGTCGATG-3’  Reverse: 5’-CAGTGCGTGTCGTGGAGT-3’ | 60 | 63 |
| hsa-miR-548ao-3p | Forward: 5’-GGGGAAAAGACCGTGACTACT-3’  Reverse: 5’-GTGCGTGTCGTGGAGTCG-3’ | 60 | 65 |

**Additional file 1: Table S4. Primers used for generating Luciferase reporters.**

| Plasmid | Primer sequence(5’-3’) | Mutated sites and locations |
| --- | --- | --- |
| pMIR-Report-Luc-TUSC3  -3UTR-WT | Forward:5’-GGACTAGTGAAGATGTGATTTGGACCATGGCA-3’  Reverse:5’-CGACGCGTGCTACTGGGCTTACTCATGTGAA-3’ | No mutations |
| pMIR-Report-Luc-TUSC3  -3UTR-a*  (hsa-miR181a-5p) | Forward:5’-TGGGATTTGCATAAAGGCCGAACCTACCATGAAG-3’  Reverse:5’-GGTTCGGCCTTTATGCAAATCCCACTTGGCTTCA-3’ | TGAATGTT GCCGAACC  (NTs 1477-1484) |
| pMIR-Report-Luc-TUSC3  -3UTR-ab*  (hsa-miR181a-5p) | Forward:5’-TGGGATTTGCATAAAGGCCGAACCTACCATGAAG-3’  Reverse:5’-GGTTCGGCCTTTATGCAAATCCCACTTGGCTTCA-3’  Forward:5’-ATATAGTTTGCTTGCACCGAACAACGTGCAAA-3’  Reverse:5’-GTTCGGTGCAAGCAAACTATATGTTTGAGCCA-3’ | TGAATGTT GCCGAACC  (NTs 1477-1484)  GAATGT CCGAAC  (NTs 3565-3570) |
| pMIR-Report-Luc-TUSC3  -3UTR-c*  (hsa-miR30e-5p) | Forward:5’-ATTTGCATAAAGTGAAGAACGCGGATGAAGATAA-3’  Reverse:5’-CCGCGTTCTTCACTTTATGCAAATCCCACTTGGC-3’ | TGTTTACC GAACGCGG  (NTs 1481-1488) |
| pMIR-Report-Luc-TUSC3  -3UTR-cd*  (hsa-miR30e-5p) | Forward:5’-ATTTGCATAAAGTGAAGAACGCGGATGAAGATAA-3’  Reverse:5’-CCGCGTTCTTCACTTTATGCAAATCCCACTTGGC-3’  Forward:5’-ATTCATGTTTTAGAGCTGTATACTCATTAGT-3’  Reverse:5’-TACAGCTCTAAAACATGAATTCATAATTAC-3’ | TGTTTACC GAACGCGG  (NTs 1481-1488)  TGTTTAC TGTATAC  (NTs 1737-1743) |
| pMIR-Report-Luc-TUSC3  -3UTR-abcd*  (hsa-miR30e-5p and  hsa-miR181a-5p) | Forward:5’-TGGGATTTGCATAAAGGCCGAACCTACCATGAAG-3’  Reverse:5’-GGTTCGGCCTTTATGCAAATCCCACTTGGCTTCA-3’  Forward:5’-ATTCATGTTTTAGAGCTGTATACTCATTAGT-3’  Reverse:5’-TACAGCTCTAAAACATGAATTCATAATTAC-3’  Forward:5’-ATATAGTTTGCTTGCACCGAACAACGTGCAAA-3’  Reverse:5’-GTTCGGTGCAAGCAAACTATATGTTTGAGCCA-3’ | TGAATGTT GCCGAACC  (NTs 1477-1484)  TGTTTAC TGTATAC  (NTs 1737-1743)  GAATGT CCGAAC  (NTs 3565-3570) |

**Nucleotides in red indicate mutated sequence at the 3’-UTR of TUSC3 to disrupt the putative miRNA-TUSC3 interactions. NTs, nucleotides.**

**Additional file 1: Table S5. Sequences of miRNA mimics and inhibitors.**

| Category | miR or anti-miR | Mature sequence | Catalog number |
| --- | --- | --- | --- |
| miRNA mimics | hsa-miR30e-5p mimics | UGUAAACAUCCUUGACUGGAAG | miR10000692-1-2 |
|  | hsa-miR181a-5p mimics | AACAUUCAACGCUGUCGGUGAGU | miR10000256-1-2 |
|  | Mimics negative control | UUUGUACUACACAAAAGUACUG | miR01101-1-2 |
| miRNA inhibitor | hsa-miR30e-5p inhibitor | UGUAAACAUCCUUGACUGGAAG | miR20000692-1-2 |
|  | hsa-miR181a-5p inhibitor | AACAUUCAACGCUGUCGGUGAGU | [miR20000256-1-2](http://www.ribobio.com/sitecn/productinfo_166930.html) |
|  | Inhibitor negative control | UUUGUACUACACAAAAGUACUG | miR02101-1-2 |

**Additional file 1: Table S6. microRNAs that were significantly up-regulated in ZR7530 following *SOX2* knockdown as detected by microarray analysis**.

| **up-regulated(SOX2-Kd/Scramble control)** | | |
| --- | --- | --- |
| miRNA | Fold change | p-value |
| hsa-miR-7-2-3p | 9.37 | 0.02 |
| hsv1-miR-H5-3p | 5.50 | 0.04 |
| hsa-miR-5007-3p | 3.66 | 0.01 |
| hsa-miR-3924 | 2.42 | 0.03 |
| hsa-miR-4305 | 2.16 | 0.0004 |
| hsa-miR-548h-3p  /hsa-miR-548z | 2.13 | 0.04 |
| hsa-miR-17-5p | 2.09 | 0.04 |
| hsa-miR-518d-5p/hsa-miR-520c-5p  /hsa-miR-526a | 1.83 | 0.02 |
