# Supplementary material for: SOX2 regulates multiple malignant processes of breast cancer development through the SOX2/miR-181a-5p, miR-30e-5p/TUSC3 axis
Source: Mol Cancer. 2017 Mar 14;16:62. doi: 10.1186/s12943-017-0632-9 (PMC5348847; doi:10.1186/s12943-017-0632-9)

**Additional file 2: Figure. S1. Construction of Luciferase reporters.** The primers used for the construction of these reporters are showed in Supplementary Table2. To obtain the pMIR-Report-TUSC33UTR-WT, a plasmid containing the wild-type 3’UTR of TUSC3 was amplified with high fidelity PCR. The PCR products were subcloned into the pMIR-Report vector (Ambion) digested with SpeI and MluI, generating pMIR-Report-Luc-TUSC3-3UTR-WT. Using overlap PCR and vector construction, we also obtained other luciferase reporters containing mutated binding sites of miR-181a-5p and miR-30e-5p and these reporters include, the reporter pMIR-Report-Luc-TUSC3-3UTR-a* containing mutated nucleotides (nucleotides 1477-1484), pMIR-Report-Luc-TUSC3-3UTR-ab* containing mutated nucleotides (nucleotides 1477-1484 and 3565-3570), pMIR-Report-Luc-TUSC3-3UTR-c* containing mutated nucleotides (nucleotides 1481-1488), pMIR-Report-Luc-TUSC3-3UTR-cd* containing mutated nucleotides (nucleotides 1481-1488 and 1737-1743), pMIR-Report-Luc-TUSC3-3UTR-abcd* (nucleotides 1477-1484, 3565-3570 and 1737-1743). Primers used for generating mutation are listed in Supplementary Table3. CMV prmt, CMV promoter. Luc, Luciferase. 3UTR, 3’ untranslated region. SV40 pA, SV40 polyA.


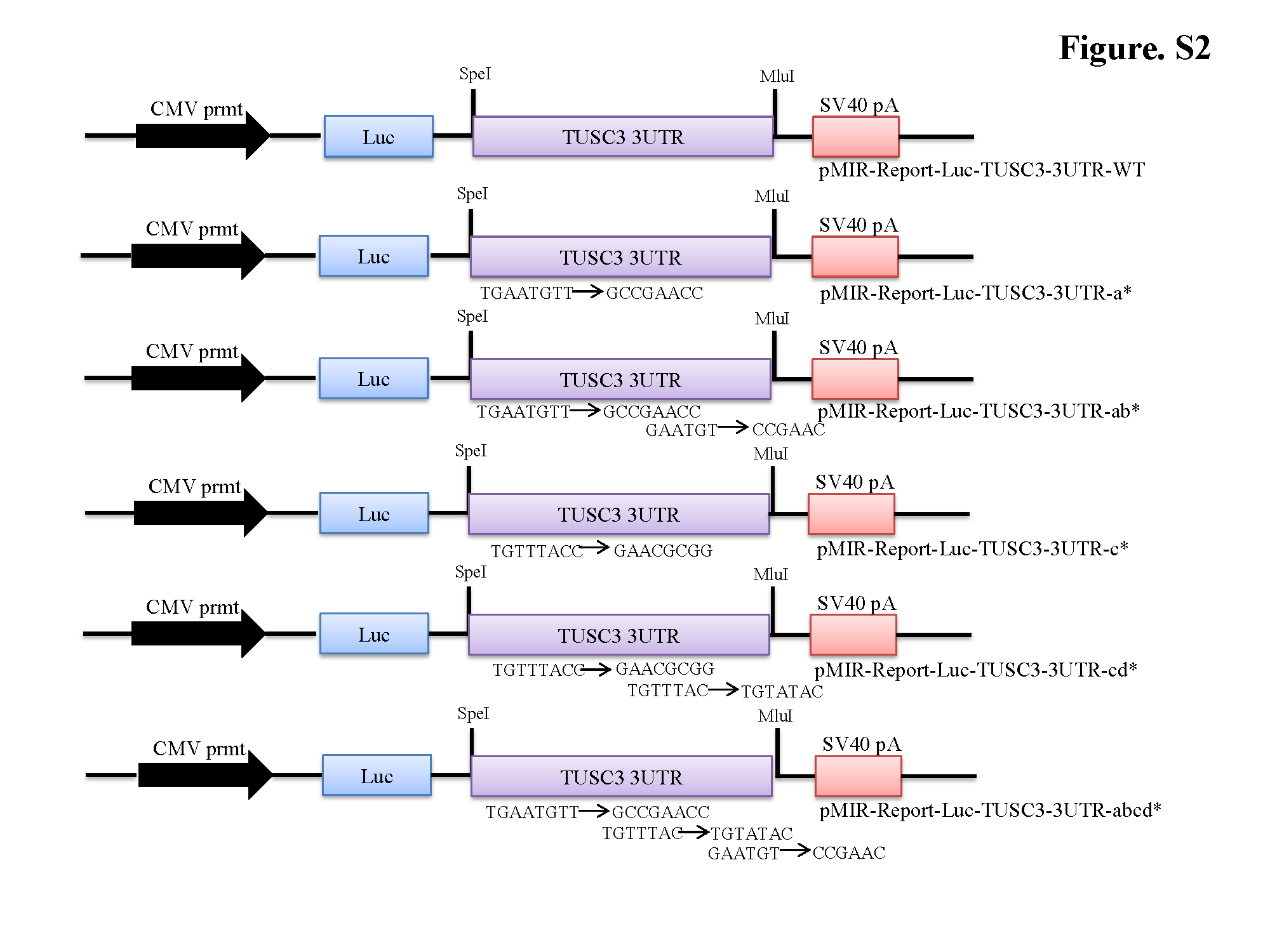

Supplement: Additional file 2: Figure S1. — Construction of Luciferase reporters. (DOC 158 kb) [file 12943_2017_632_MOESM2_ESM.doc]
